# Integration of MRI-Based Radiomics Features, Clinicopathological Characteristics, and Blood Parameters: A Nomogram Model for Predicting Clinical Outcome in Nasopharyngeal Carcinoma

**DOI:** 10.3389/fonc.2022.815952

**Published:** 2022-03-02

**Authors:** Zeng-Yi Fang, Ke-Zhen Li, Man Yang, Yu-Rou Che, Li-Ping Luo, Zi-Fei Wu, Ming-Quan Gao, Chuan Wu, Cheng Luo, Xin Lai, Yi-Yao Zhang, Mei Wang, Zhu Xu, Si-Ming Li, Jie-Ke Liu, Peng Zhou, Wei-Dong Wang

**Affiliations:** ^1^ Department of Radiation Oncology, Sichuan Cancer Hospital and Institute, Chengdu, China; ^2^ Department of Oncology, School of Clinical Medicine, Southwest Medical University, Luzhou, China; ^3^ Radiation Oncology, Key Laboratory of Sichuan Province, Chengdu, China; ^4^ School of Medicine, University of Electronic Science and Technology of China, Chengdu, China

**Keywords:** radiomics, progression-free survival, nasopharyngeal carcinoma, Ki-67, blood parameters

## Abstract

**Purpose:**

This study aimed to develop a nomogram model based on multiparametric magnetic resonance imaging (MRI) radiomics features, clinicopathological characteristics, and blood parameters to predict the progression-free survival (PFS) of patients with nasopharyngeal carcinoma (NPC).

**Methods:**

A total of 462 patients with pathologically confirmed nonkeratinizing NPC treated at Sichuan Cancer Hospital were recruited from 2015 to 2019 and divided into training and validation cohorts at a ratio of 7:3. The least absolute shrinkage and selection operator (LASSO) algorithm was used for radiomics feature dimension reduction and screening in the training cohort. Rad-score, age, sex, smoking and drinking habits, Ki-67, monocytes, monocyte ratio, and mean corpuscular volume were incorporated into a multivariate Cox proportional risk regression model to build a multifactorial nomogram. The concordance index (C-index) and decision curve analysis (DCA) were applied to estimate its efficacy.

**Results:**

Nine significant features associated with PFS were selected by LASSO and used to calculate the rad-score of each patient. The rad-score was verified as an independent prognostic factor for PFS in NPC. The survival analysis showed that those with lower rad-scores had longer PFS in both cohorts (*p* < 0.05). Compared with the tumor–node–metastasis staging system, the multifactorial nomogram had higher C-indexes (training cohorts: 0.819 vs. 0.610; validation cohorts: 0.820 vs. 0.602). Moreover, the DCA curve showed that this model could better predict progression within 50% threshold probability.

**Conclusion:**

A nomogram that combined MRI-based radiomics with clinicopathological characteristics and blood parameters improved the ability to predict progression in patients with NPC.

## 1 Introduction

Nasopharyngeal carcinoma (NPC) is a malignant tumor in the mucous membrane of the nasopharynx. The incidence and mortality of NPC vary in regional distribution, especially in Southeast Asia (1–3). Although intensity-modulated radiotherapy (IMRT) significantly improved the prognosis of NPC, some patients still experience progression (4, 5). At present, the risk assessment of NPC is mainly determined by the tumor–node–metastasis (TNM) staging system, which only has 61% accuracy for predicting the local recurrence of NPC (6). While it incorporates local tumor invasion, positive lymph nodes, and distant metastases, TNM cannot explain the temporal and spatial heterogeneity or changes in the internal and external environments of tumor cells. Plasma Epstein–Barr virus (EBV) DNA, which may affect the growth and apoptosis of the NPC cell line, has been used as an independent prognostic marker in endemic areas, but the detection rate of EBV is low in nonendemic areas (7, 8). Therefore, it is urgent to identify more representative and comprehensive biomarkers to predict NPC prognosis.

Many studies reported that a large number of clinical biomarkers such as monocytes (MONO), mean corpuscular volume (MCV), and Ki-67 expression are associated with the tumor microenvironment and tumor immune escape (9–11). There are no regional differences in the expression of these markers. Beyond these biomarkers, the emerging field of radiomics is supposed to be a bridge between medical imaging and clinical medicine (12). Radiomics features are used for tumor diagnosis, phenotype, and prognosis (13–15). By extracting innumerable quantitative imaging features, the differences in tumor heterogeneity and microenvironment may be explained. Some recent studies showed that magnetic resonance imaging (MRI) radiomics were significantly associated with NPC prognosis (16–18). However, no publications integrated blood parameters, Ki-67, and MRI radiomics to predict progression-free survival (PFS) in patients with NPC.

We built and validated a nomogram prediction model based on MRI, clinicopathological parameters, and blood parameters to visually demonstrate the PFS of NPC and guide clinical diagnosis and treatment.

## 2 Materials and Methods

### 2.1 Patients

**Figure 1:**
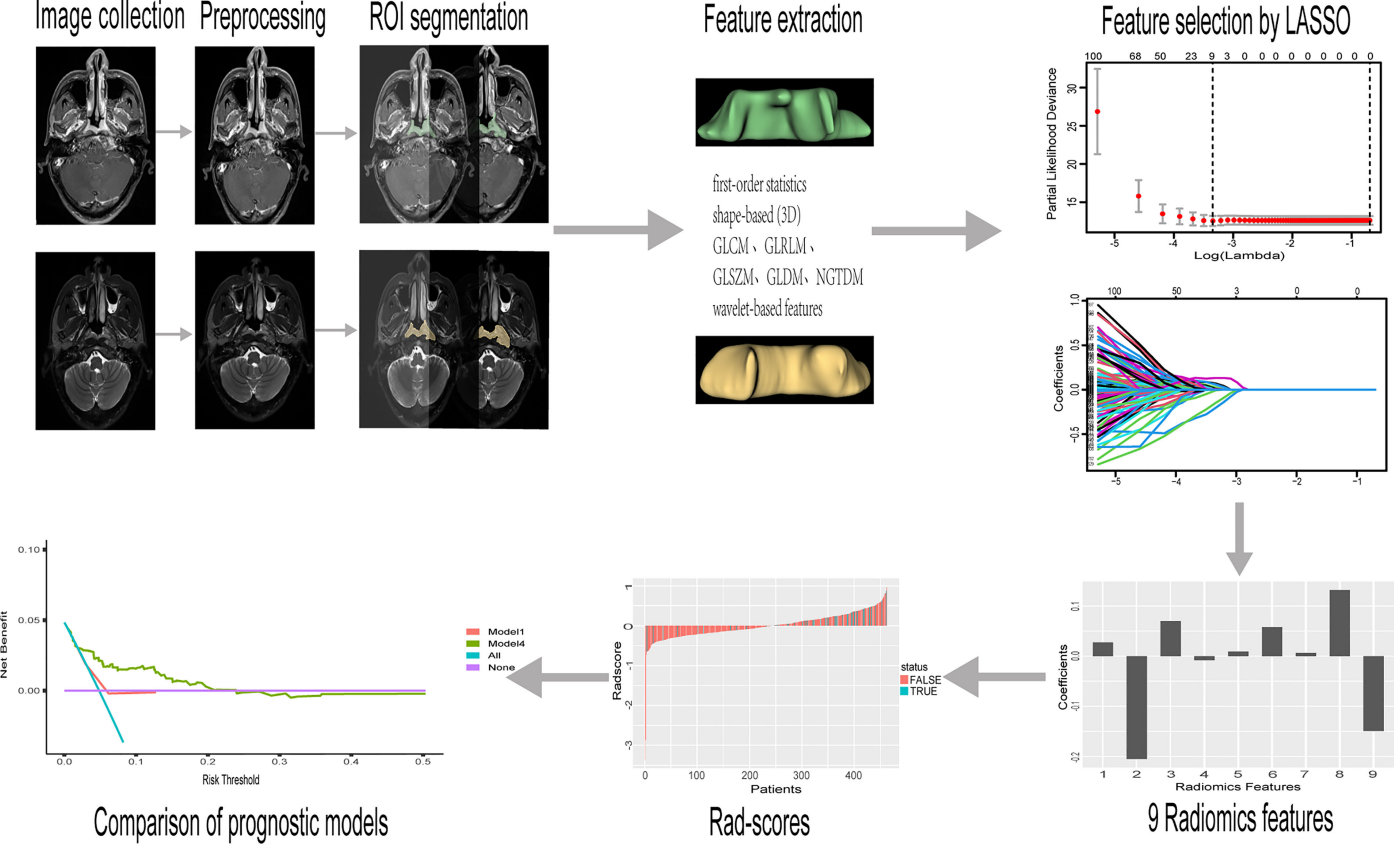
The workflow of MRI-based radiomic analysis. After manual tumor segementation, 2074 features of each patients were extracted. Radiomics features selection by the LASSO algorithm. These selected features were linearly fitted according to the weights of the coefficients to calculate the rad-score. Decision curve analysis (DCA) compared the net benefit rate between the TNM stage system (Model 1 ) with our nomogram model (Model 4).

Data from patients treated in Sichuan Cancer Hospital from January 2015 to December 2019 were reviewed. The inclusion and exclusion criteria are presented in the **Supplementary Data**. The study workflow is displayed in **Figure 1**. A total of 462 patients were included and randomly divided into a training cohort (*n* = 323) and validation cohort (*n* = 139) at a 7:3 ratio. The method and criteria of Ki-67 scoring are detailed in the **Supplementary Data**. Clinical data (age, gender, smoking and drinking habits, TNM, plasma EBV DNA) and blood parameters were collected. All patients were restaged according to the 8th Edition American Joint Committee on Cancer TNM Staging System (19).

### 2.2 Treatment

#### 2.2.1 Radiotherapy

All patients underwent IMRT. Delineation of the target area and organs at risk were based on ICRU reports 50 and 62. The prescribed doses for the target area were GTVnx 66–76 Gy, GTVnd 66–70 Gy, CTV1 60–62 Gy, CTV2 50–56 Gy, and CTVnd 50–56 Gy (28–33 fractions).

#### 2.2.2 Chemotherapy

Patients with stage II (*n* = 23) underwent concurrent chemoradiotherapy (CCRT). Those with stages III–IV (*n* = 439) were treated with two cycles of induction chemotherapy (IC) followed by CCRT. The IC drugs were cisplatin (75 mg/m^2^, d1–3) plus paclitaxel (135 mg/m^2^, d1) every 3 weeks for two cycles. The CCRT drug was cisplatin (75 mg/m^2^, d1–3) given every 3 weeks.

### 2.3 Follow-Up

After patients completed all treatments, they were followed-up every 3 months in the first 2 years, every 6 months in years 3–5, and annually thereafter. The review items included blood parameters, nasopharyngeal MRI, chest computed tomography, abdominal ultrasonography, or isotope bone scanning, and each review item was determined according to the specific situation of the patient. PFS was set as the primary endpoint.

### 2.4 MRI Acquisition and Image Preprocessing

The pretreatment MRI parameters are listed in the **Supplementary Data**. To avoid inhomogeneity due to different MRI devices, two image preprocessing steps were applied. First, we used the N4ITK algorithm to remove bias field artifacts (20). Second, the intensity range was adjusted from 0 to 255. In addition to the original images, the Gaussian Laplace filter with sigma values of 4 and 5 mm was used to reconstruct the images, and the features of the multiscale resolution were extracted (21, 22). Preprocessing was performed in the SimpleITK 2.0.2, which is an open-source platform for Python 3.8.5 (www.python.org).

### 2.5 Image Segmentation

We used 3D Slicer 4.11 software (open source and multiplatform software; www.slicer.org) for manual segmentation (23). A radiologist with 20 years of experience delineated the region of interest (ROI), which refers to the margin of the nasopharyngeal tumor at each level on axial CET1-w and T2-w images.

### 2.6 Extraction of Radiomics Features

A total of 1,037 radiomics features were obtained by SlicerRadiomics (an extension for 3D Slicer 4.11 that encapsulates pyradiomics library) from axial CET1-w and T2-w images, respectively. Features of different categories were considered: first-order statistics, shape-based (3D), gray-level co-occurrence matrix (GLCM), gray-level run length matrix (GLRLM), gray-level size zone matrix (GLSZM), gray-level dependence matrix (GLDM), neighborhood gray tone difference matrix (NGTDM), and wavelet-based features.

### 2.7 Postprocessing of Radiomics Features and Building of Radiomics Signature

To ensure the comparability of different features, Z-score normalization was performed to unify data from different levels into the same level. Feature selection was conducted in the training cohort (*n* = 323). We used the least absolute shrinkage and selection operator (LASSO) algorithm for feature dimension reduction and screening. LASSO attempts to shrink some coefficients of the models and sets others to zero, but it may lead to overfitting, so we added a 10-fold cross-validation. Nine noteworthy features were selected. These features were linearly fitted according to the weights of their coefficients; for each patient, the rad-score was calculated. The rad-score was then used to build the radiomics signature.

### 2.8 Radiomics Survival Model Development and Validation

To find the rad-score cutoff with the best sensitivity and specificity, we generated a receiver operating characteristic curve (ROC) using data from the training cohort. To explore the potential association between radiomics features and PFS, we separated patients in both cohorts into high- and low-risk groups based on the cutoff value of rad-scores (patients below this cutoff value were considered low risk). Kaplan–Meier survival analysis was used to identify PFS differences in both cohorts.

### 2.9 Evaluation and Comparison of the Multifactorial Prognostic Nomogram Model

Four models were set up to compare the prognostic efficacy (model 1: clinical stage; model 2: radiomics; model 3: clinical stage + rad-score; model 4: clinical data + rad-score). The concordance index (C-index) was used to evaluate univariate or multivariate Cox models. A nomogram was built to visualize the results of the best prediction model in the training cohort using the R software (version 4.1.0). We evaluated the uniformity of the nomogram by plotting 3- and 5-year calibration curves. Decision curve analysis (DCA) was performed to compare the net benefit rate between the TNM stage system and this nomogram for predicting prognosis.

### 2.10 Statistical Analysis

Statistical analyses were performed with the R software (version 4.1.0; www.r-project.org), SPSS (SPSS version 20.0, IBM Corp, Armonk, NY, USA), and Python 3.8.5. Clinical data were compared between the training and validation cohorts with Independent samples *t*-tests, Mann–Whitney *U* tests, or Chi-square tests. Missing data was processed using the “miceforest” package from Python. Several R packages were employed: LASSO in the “glmnet” package was used to select radiomics features. Kaplan–Meier survival, Cox proportional hazard regression, and C-index were calculated by the “survival” and “rms” packages. DCA was performed with the “ggDCA” package. The “pROC” and “ggplot2” packages were applied to generate the ROC curve and rad-score histogram, respectively. For all statistical tests, differences were considered significant at *p* < 0.05.

## 3 Results

### 3.1 Clinical Parameters

This retrospective study included 462 patients with pathologically confirmed nonkeratinizing NPC who were treated at Sichuan Cancer Hospital between January 2015 and December 2019. The clinical parameters of all patients in the training and validation cohorts are listed in **Table 1**. The median age was 49 years (range: 12–82 years), with 329 men and 133 women. The numbers of patients with each clinical stage were 0, 23, 193, 226, and 20 for stages I, II, III, IVA, and IVB, respectively. The Ki-67 cutoff value from the ROC curve was 37.5% (range: 3%–90%). The cutoff value for classifying EBV infection status was 400 copies/ml (negative: <400 copies/ml; positive: ≥400 copies/ml). A total of 330 patients who met the inclusion criteria underwent plasma EBV DNA tests before treatment, and 112 were positive. Among them, there were 2 cases of stage II, 24 cases of stage III, and 86 cases of stage IV. The interpolation of EBV DNA missing data was performed using the multiple substitutions in chained equations (MICE) method of random forest. The **Supplementary Data** detail the results after interpolating EBV DNA. The median PFS was 33.15 months (0.6–76.2 months) for all patients; 45 patients progressed, including 23 deaths, 14 distant metastases, and 8 recurrences.

**Table 1:** Clinical parameters of patients in the training and validation cohorts.

	Training cohort (*n* = 323)	Validation cohort (*n* = 139)	*p*-value
Gender			0.652
Male	228 (70.6%)	101 (72.7%)	
Female	95 (29.4%)	38 (27.3%)	
Age (years)			0.949
≥49	167 (51.7%)	70 (50.4%)	
<49	156 (48.3%)	69 (49.6%)	
Overall stage			0.000
I	0	0	
II	18 (5.6%)	5 (3.6%)	
III	132 (40.9%)	61 (43.9%)	
IVA	158 (48.9%)	68 (48.9%)	
IVB	15 (4.6%)	5 (3.6%)	
T stage			0.000
T1	20 (6.2%)	9 (6.5%)	
T2	77 (23.8%)	36 (25.9%)	
T3	118 (36.5%)	49 (35.3%)	
T4	108 (33.5%)	45 (32.3%)	
N stage			0.000
N0	5 (1.5%)	4 (2.9%)	
N1	42 (13.0%)	16 (11.5%)	
N2	191 (59.1%)	78 (56.1%)	
N3	85 (26.4%)	41 (29.5%)	
M stage			0.000
M0	308 (95.4%)	134 (96.4%)	
M1	15 (4.6%)	5 (3.6%)	
Smoking			0.036
No	201 (62.2%)	72 (51.8%)	
Yes	122 (37.8%)	67 (48.2%)	
Drinking			0.111
No	246 (76.2%)	96 (69.1%)	
Yes	77 (23.8%)	43 (30.9%)	
Ki-67 (%)			0.680
≥37.5	238 (73.7%)	98 (70.5%)	
<37.5	85 (26.3%)	41 (29.5%)	
EBV			0.664
Positive	76 (23.5%)	36 (25.9%)	
Negative	153 (47.4%)	65 (46.8%)	
None	94 (29.1%)	38 (27.3%)	

Statistical comparisons between the training and validation cohorts were performed with Independent samples t-tests, Mann–Whitney U tests, or Chi-square tests. p-values <0.05 were considered statistically significant.

EBV, Epstein–Barr virus.

### 3.2 Blood Parameters

All blood parameters in the training and validation cohorts are shown in **Table 2**. The cutoff values identified with ROC curves are shown in the **Supplementary Data**, as are the values of the areas under curve (AUCs) for blood parameters. The highest AUC values were found for MONO, MONO%, and MCV, which were 0.637, 0.626, and 0.568, respectively. These were incorporated into model 4.

**Table 2:** Blood parameters in the training and validation cohorts.

	Training cohort (*n* = 323)	Validation cohort (*n* = 139)	*p*-value
WBC (10^9^/L)			0.439
≥6.695	111 (34.4%)	45 (32.4%)	
<6.695	212 (65.6%)	94 (67.6%)	
GR (10^9^/L)			0.833
≥3.105	230 (71.2%)	100 (71.9%)	
<3.105	93 (28.8%)	39 (28.1%)	
LYMPH (10^9^/L)			0.636
≥1.960	77 (23.8%)	31 (22.3%)	
<1.960	246 (76.2%)	108 (77.7%)	
MONO (10^9^/L)			0.643
≥0.385	150 (46.4%)	59 (42.4%)	
<0.385	173 (53.6%)	80 (57.6%)	
EO (10^9^/L)			0.841
≥0.175	95 (29.4%)	38 (27.3%)	
<0.175	228 (70.6%)	101 (72.7%)	
BASO (10^9^/L)			0.877
≥0.035	76 (23.5%)	35 (25.2%)	
<0.035	247 (76.5%)	104 (74.8%)	
GR%			0.868
≥69.150	82 (25.4%)	47 (33.8%)	
<69.150	241 (74.6%)	92 (66.2%)	
LYMPH%			0.944
≥29.850	97 (30%)	46 (33.1%)	
<29.850	226 (70%)	93 (66.9%)	
MONO%			0.595
≥5.950	168 (52%)	75 (54%)	
<5.950	155 (48%)	64 (46%)	
EO%			0.856
≥2.050	156 (48.3%)	64 (46%)	
<2.050	167 (51.7%)	75 (54%)	
BASO%			0.856
≥0.350	224 (69.3%)	89 (64%)	
<0.350	99 (30.7%)	50 (36%)	
RBC (10^12^/L)			0.262
≥4.685	132 (40.9%)	62 (44.6%)	
<4.685	191 (59.1%)	77 (55.4%)	
HGB (g/L)			0.616
≥125	266 (82.4%)	112 (80.6%)	
<125	57 (17.6%)	27 (19.4%)	
HCT			0.899
≥44.050	114 (35.3%)	50 (36%)	
<44.050	209 (64.7%)	89 (64%)	
MCV (fl)			0.057
≥95.650	106 (32.8%)	39 (28.1%)	
<95.650	217 (67.2%)	100 (71.9%)	
MCH (pg)			0.424
≥32.750	34 (10.5%)	10 (7.2%)	
<32.750	289 (89.5%)	129 (92.8%)	
MCHC (g/L)			0.132
≥337.500	40 (12.4%)	23 (16.5%)	
<337.500	283 (87.6%)	116 (83.5%)	
RDW_CV			0.454
≥12.950	210 (65%)	94 (67.6%)	
<12.950	113 (35%)	45 (32.4%)	
RDW_SD (fl)			0.524
≥42.850	176 (54.5%)	71 (51.1%)	
<42.850	147 (45.5%)	68 (48.9%)	
PLT (10^9^/L)			0.698
≥191	182 (56.3%)	82 (59%)	
<191	141 (43.7%)	57 (41%)	
MPV (fl)			0.099
≥12.250	98 (30.3%)	28 (20.1%)	
<12.250	225 (69.7%)	111 (79.9%)	
PDW			0.615
≥16.050	254 (78.6%)	106 (76.3%)	
<16.050	69 (21.4%)	33 (23.7%)	
PCT			0.807
≥0.245	110 (34.1%)	47 (33.8%)	
<0.245	213 (65.9%)	92 (66.2%)	
NLR			0.991
≥2.026	232 (71.8%)	94 (67.6%)	
<2.026	91 (28.2%)	45 (32.4%)	
PLR			0.990
≥132.020	150 (46.4%)	59 (42.4%)	
<132.020	173 (53.6%)	80 (57.6%)	
LMR			0.601
≥4.822	113 (35%)	50 (36%)	
<4.822	210 (65%)	89 (64%)	

Statistical comparisons between the training and validation cohorts were performed with Independent samples t-tests, Mann–Whitney U tests, or Chi-square tests. p-values of <0.05 were considered statistically significant.

BASO, basophils; BASO%, ratio of basophils; EO, eosinophils; EO%, ratio of eosinophils; GR, neutrophilic granulocytes; GR%, ratio of neutrophilic granulocytes; HCT, hematocrit; HGB, hemoglobin; LMR, lymphocyte-to-monocyte ratio; LYMPH, lymphocytes; LYMPH%, ratio of lymphocytes; MCH, mean corpuscular hemoglobin; MCHC, mean corpuscular hemoglobin concentration; MCV, mean corpuscular volume; MONO, monocytes; MONO%, ratio of monocytes; MPV, mean platelet volume; NLR, neutrophil-to-lymphocyte ratio; PCT, plateletcrit; PDW, platelet distribution width; PLR, platelet to lymphocyte ratio; PLT, platelets; RBC, red blood cells; RDW-CV, variation of RBC distribution width; RDW-SD, standard deviation of RBC distribution width; WBC, white blood cells.

### 3.3 Radiomics Signature Development

**Figure 2:**
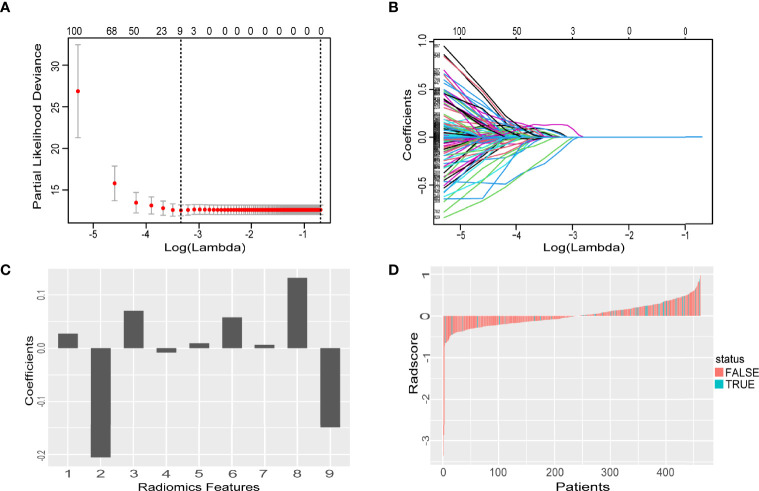
Radiomics feature selection using the LASSO algorithm. **(A)** Used the 1O-fold cross validation to identify the optimal penalization coefficient lambda the minimum was 0.000577, with log (λ) = -3.238. **(B)** The model coefficient trendlines of radiomics features. **(C)** The histogram of coefficients with 9 features. **(D)** Rad-score for each patient. Red bars show scores for patients who survived without progression, while blue bars show scores for patients who happened progression, metastasis or died.

In total, 2,074 features were obtained from each ROI. The final nine key features were selected by LASSO ((1) CET1-w_Log-sigma-5-0-mm_glrlm_HighGrayLevelRunE-mphasis; (2) CET1-w_wavelet-LLH_glcm_ClusterShade; (3) CET1-w_wavelet-LLH_gl-szm_GrayLevelNonUniformity; (4) CET1-w_wavelet-HHL_glcm_Correlation; (5) CE-T1-w_wavelet-HHH_firstorder_Mean; (6) CET1-w_wavelet-HHH_gldm_LargeDepen-denceHighGrayLevelEmphasis; (7) T2-w_log-sigma-4-0-mm_firstorder_Maximum; (8) T2-w_wavelet-HHL_firstorder_Maximum; (9) T2-w_wavelet-HHL_glcm_InverseVar-iance). The rad-score was calculated for each patient according to the weights of their coefficients. The formula for calculating the rad-score is detailed in the **Supplementary Data**. The features selected by LASSO and the histogram of every patient’s rad-score are shown in **Figures 2A–D**.

### 3.4 Model Predictions and Comparison

**Figure 3:**
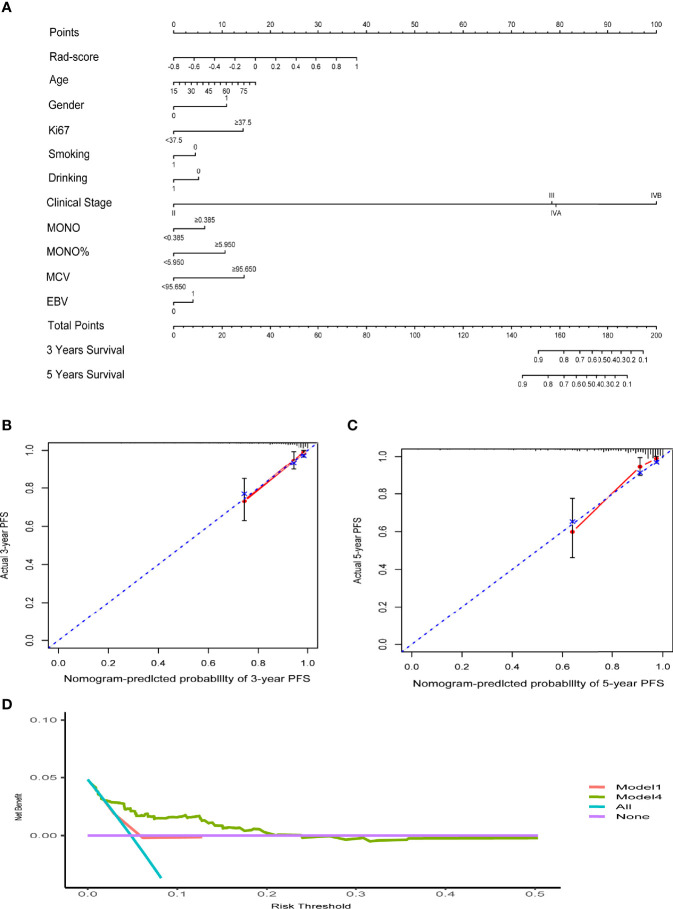
**(A)** The nomogram of clinical data and rad-score. **(B, C)** The calibration curves of the nomogram. **(D)** Decision curve analysis for Model4 and Model1. The y-axis measures the net benefit. The red line represents Model 1 (clinical stage). The green line represents Model 4 (clinical data and rad-score). The blue line assumes that all patients progress. The purple line indicates that no progression is assumed in all patients.

The C-indexes of the four models are listed in **Table 3**. The C-index of model 2 was significantly higher than that of model 1 in both cohorts, which suggested that the predictive effect of radiomics may surpass that of the TNM stage system. Moreover, when comparing models 1 and 3, we found that model 3 that included the rad-score could remarkably predict the prognostic potency of the clinical stage. Model 4 integrating clinical data and radiomics had the best probability that the predicted results were consistent with the observed results (C-index of training and validation: 0.823 (95% CI: 0.745–0.901) vs. 0.812 (95% CI: 0.693–0.930)). The nomogram of model 4 is also shown in **Figure 3A**. Notably, the calibration curves of 3–5 years were very close to the diagonal line (**Figures 3B, C**). The DCA results for models 4 and 1 are presented in **Figure 3D**, confirming the remarkable effectiveness of model 4.

**Table 3:** C-indexes of the four models.

Models	Training cohort (*n* = 323)	Validation cohort (*n* = 139)
**1 Clinical stage**	0.610 (95% CI: 0.507–0.714)	0.602 (95% CI: 0.474–0.729)
**2 Radiomics**	0.814 (95% CI: 0.746–0.882)	0.728 (95% CI: 0.618–0.838)
**3 Clinical stage + rad-score**	0.708 (95% CI: 0.602–0.814)	0.681 (95% CI: 0.562–0.801)
**4 Clinical data + rad-score**	0.823 (95% CI: 0.745–0.901)	0.812 (95% CI: 0.693–0.930)

Clinical data included gender, age, Ki-67, smoking and drinking habits, clinical stage, MONO, MONO%, MCV, and EBV DNA.

CI, confidence interval.

### 3.5 Kaplan–Meier Survival Analysis

**Figure 4:**
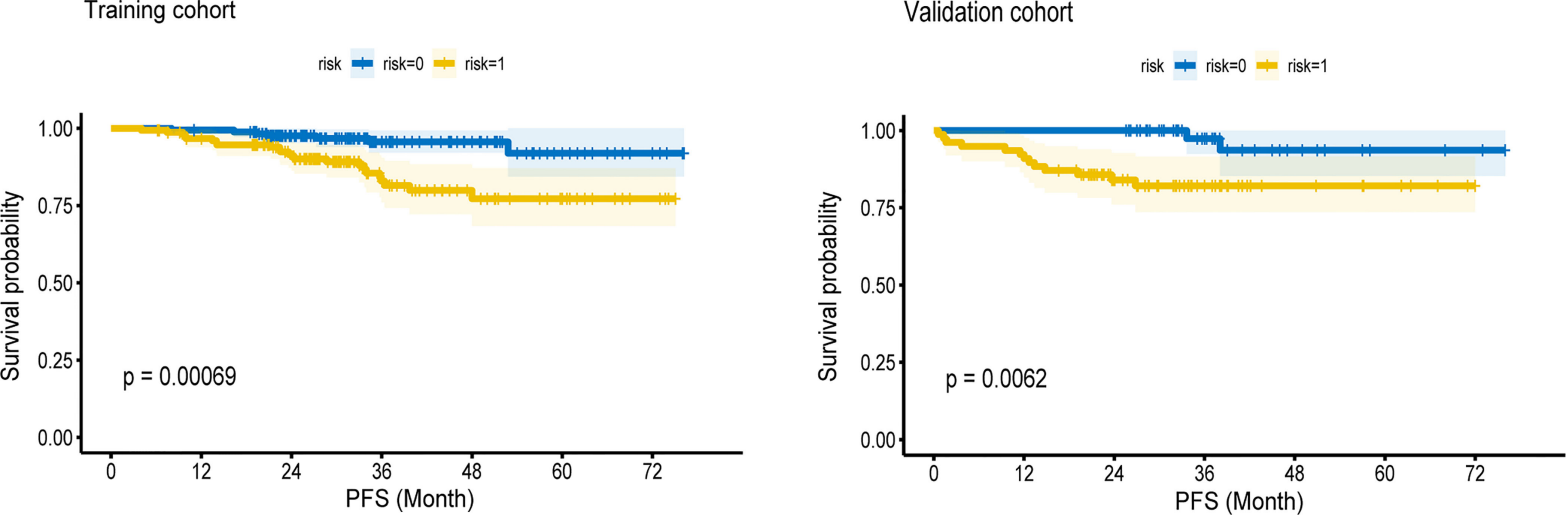
The Kaplan—Meier survival curves of high-risk and low-risk groups in the training cohort and validation cohort. In both cohorts, the low-risk group had longer PFS (P < 0.05).

Kaplan–Meier survival curves were drawn based on rad-scores. The cutoff value from the ROC curve was −0.021. A rad-score below this cutoff was considered low risk. In both cohorts, the low-risk group had significantly longer PFS (*p* < 0.05) (**Figure 4**).

## 4 Discussion

We designed this study to build and validate multimodal information from MRI-based radiomics as an effective way to estimate PFS in patients with NPC. Our findings suggested that the multidimensional nomogram combining clinicopathological characteristics, blood parameters, and rad-score was superior to the prediction performance of the TNM staging system. Moreover, using the cutoff value of the rad-score, patients could be distinguished into high- and low-risk groups, and the latter had longer PFS.

In recent years, a growing number of studies have reported that MRI radiomics features can better reflect prognostic information for NPC because they may explain the inherent temporal or spatial heterogeneity of tumors on imaging (24–26). Kim et al. studied CET1-w and T2-w MRI images of 81 patients with NPC and conclude that MR-based radiomics features showed better performance than the TNM staging system and clinical variables. Their model combined radiomics features with TNM stage and clinical variables to provide the highest AUC values (27). Shen et al. found that a model that incorporated radiomics, clinical stage, and EBV DNA status from 327 nonmetastatic NPC, yielded a high C-index in two cohorts [0.805 (95% CI: 0.768–0.841) vs. 0.874 (95% CI: 0.861–0.877)] (28). In our study, the model that integrated clinical data with radiomics features also performed best; the C-index values of model 4 were 0.823 (95% CI: 0.745–0.901) in the training cohort and 0.812 (95% CI: 0.693–0.930) in the validation cohort. The C-index of our validation cohort was lower than that reported in the study by Shen. A possible explanation may be that we included metastatic NPC patients and had a larger sample size, which may have improved the generalizability of the prediction model. Compared with other research, the parameters included in our model are more universal, without regional differences, so the model has a higher degree of applicability. Based on our nomogram, the probabilities of 3- and 5-year PFS of a given patient can be visually and easily estimated by using the corresponding parameters measured before treatment. If patients with short PFS are identified as early as possible, clinicians can enhance treatment without increasing side effects (e.g., by combining targeted treatment or immunotherapy), pay attention to adverse prognostic factors, and ensure an adequate follow-up period to reduce the risk of disease progression. Conversely, for patients with a high probability of 3- and 5-year PFS predicted by the model, it may be possible to reduce the drug dose and mitigate side effects.

However, several key aspects need to be considered when developing a clinical radiomics predictive model. Firstly, since plasma EBV DNA is used as an independent prognostic marker in endemic areas, many studies have incorporated it in nomogram construction. The MICE algorithm has the benefit of fast and efficient memory, which makes the results reliable even with missing ENV DNA data. Currently, we are expanding the sample size or conducting multicenter studies to address this issue. Compared with plasma EBV DNA, radiomics features are more advanced and accurate in predicting prognosis (29). One study reported that EBV DNA can induce monocytes to produce interluekin-10, which leads to immune escape (30). Based on this, we collected easily obtainable blood parameters from NPC, expecting to find stable markers and incorporate them into the radiomics nomogram. After drawing the ROC curve for blood parameters, we found that monocytes had the best sensitivity and specificity. Two retrospective studies validated age, gender, Ki-67, and smoking and drinking habits as independent prognostic factors for NPC (11, 31). Our results showed the model integrating clinical data and the rad-score was more useful than those only using radiomics features.

Although we successfully demonstrated the utility of radiomics data for predicting PFS in patients with NPC, this study has three major limitations. First, this was a single-center retrospective study, so the results may not readily be applicable to other situations and prospective multicenter studies are needed to confirm our findings. Second, we selected patients according to strict inclusion criteria, which may have introduced selection bias. Third, our study only focused on PFS at 3 and 5 years. In the future, we will investigate the long-term overall survival of NPC and pay more attention to predicting long-term quality of life using imaging radiomics.

In conclusion, we established an effective clinical-radiomics nomogram based on MRI findings and several clinical, pathological, and blood factors. This approach is noninvasive, visualizable, and individualized and has great potential in predicting NPC prognosis and treatment. Moreover, we further confirmed that radiomics features were independent prognostic factors for NPC.

## Data Availability Statement

The original contributions presented in the study are included in the article/**Supplementary Material**. Further inquiries can be directed to the corresponding author.

## Ethics Statement

The studies involving human participants were reviewed and approved by the ethics committee of Sichuan Cancer Hospital. Written informed consent from the participants’ legal guardian/next of kin was not required to participate in this study in accordance with the national legislation and the institutional requirements.

## Author Contributions

Z-YF and W-DW: study design, statistical analysis, and wrote the manuscript. K-ZL, MY, and Y-RC: clinical data collection. L-PL, Z-FW, M-QG, CW, J-KL, and PZ: collection of images. CL, XL, Y-YZ, MW, ZX, and S-ML: collection of blood parameters. W-DW: ROI segmentation and manuscript modification and enrichment. All authors contributed to manuscript revision and read and approved the submitted version.

## Funding

This study received funding from the National Key Research & Development Program of China (2017YFC0113904), the Sichuan Key Research & Development Project (2017SZ0004), and the Chengdu Technology Innovation R&D Project (2021YF0501659SN).

## Conflict of Interest

The authors declare that the research was conducted in the absence of any commercial or financial relationships that could be construed as a potential conflict of interest.

## Publisher’s Note

All claims expressed in this article are solely those of the authors and do not necessarily represent those of their affiliated organizations, or those of the publisher, the editors and the reviewers. Any product that may be evaluated in this article, or claim that may be made by its manufacturer, is not guaranteed or endorsed by the publisher.

## Abbreviations

MRI, multiparametric magnetic resonance imaging; PFS, progression-free survival; NPC, nasopharyngeal carcinoma; LASSO, least absolute shrinkage and selection operator algorithm; MONO, monocytes; MONO%, monocyte ratio; MCV, mean corpuscular volume; C-index, concordance index; DCA, decision curve analysis; IMRT, intensity-modulated radiotherapy; TNM, tumor–node–metastasis staging system; EBV, Epstein–Barr virus; CCRT, concurrent chemoradiotherapy; IC, induction chemotherapy; AC, adjuvant chemotherapy; GLCM, gray-level co-occurrence matrix; GLRLM, gray-level run length matrix; GLSZM, gray-level size zone matrix; GLDM, gray-level dependence matrix; NGTDM, neighborhood gray tone difference matrix; ROI, region of interest; ROC, receiver operating characteristic curve
