# Supplementary material for: Integration of MRI-Based Radiomics Features, Clinicopathological Characteristics, and Blood Parameters: A Nomogram Model for Predicting Clinical Outcome in Nasopharyngeal Carcinoma
Source: Front Oncol. 2022 Mar 2;12:815952. doi: 10.3389/fonc.2022.815952 (PMC8924617; doi:10.3389/fonc.2022.815952)
Supplement: Supplementary file 1 [file DataSheet_1.docx]

Supplementary Data

Methods 1: Inclusion and exclusion criteria for the present study.

Methods 2: The method and criteria of calculating Ki-67 scoring

Methods 3: Magnetic resonance image acquisition parameters used in the present study.

Methods 4: Plasma EBV DNA

Figure 1: The ROC curves of blood parameters.

Table 1: The values of area under curve from blood parameters.

Methods 5: The formula for calculating the rad-score.

# Methods 1 Inclusion and exclusion criteria for the present study

The inclusion criteria were: (1) histologically confirmed non-keratinizing nasopharyngeal carcinoma; (2) received pre-treatment MRI; (3) treated by intensity-modulated radiotherapy (IMGT); (4) received concurrent chemotherapy or Induction chemotherapy.

The exclusion criteria were: (1) received chemotherapy or radiotherapy before; (2) with contraindications for MRI; (3) diagnosed other malignancies.

# Methods 2 The method and criteria of calculating Ki-67 scoring

Using immunohistochemical staining, nasopharyngeal carcinoma tissues were fixed with formalin, dehydrated and paraffin-embedded into blocks, cut into 4-μm serial sections using a Leica HistoCore BIOCUT tissue slicer, dewaxed, hydrated, peroxidase blocked, antigen repaired, and rinsed multiple times with PBS. The primary anti-Ki-67 antibody (1:600) was added dropwise, incubated overnight at 4℃, rinsed with PBS solution, and then goat anti-mouse IgG (1:1000) was added dropwise. After incubation at 25℃ for 15 minutes, the slides were rinsed with PBS before color development, re-staining, and sealed.

Using a double-blind method, two senior pathologists separately assessed the positive cell rate under a microscope. Ki-67 was localized in the cell nuclei as brown particles. Ten high-power fields were randomly observed, 100 cells were counted in each field, and the mean value of positive cells among tumor cells in these fields was calculated as the Ki-67 score. (Percentage of positive cells = number of positive cells/total cell count × 100%)

# Methods 3 Magnetic resonance image acquisition parameters used in the present study

MRI Acquisition Parameters

All patients underwent pretreatment MRI.

The 1.5T Siemens MRI parameters were as follows: axial T2-weighted spin-echo images (repetition time [TR]/echo time [TE]: 3925/76ms, field of view [FOV] read=250mm, slice thickness =3 mm, averages=2, spacing between slices=3.3mm) and axial contrast-enhanced T1-weighted spin-echo images (TR/TE: 750/16ms, FOV read=250mm, slice thickness = 3mm, averages=2, spacing between slices=3.3mm).

The 3.0T Siemens MRI acquisition parameters were as follows: axial T2-weighted spin-echo images (TR/TE: 3720/80ms, FOV read= 250mm, slice thickness = 3 mm, averages=2, spacing between slices=3.3mm) and axial contrast-enhanced T1-weighted spin-echo images (TR/TE: 750/11ms, FOV read= 250mm, slice thickness =3mm, averages=2, spacing between slices=3.3mm).

# Methods 4 Plasma EBV DNA

The copy of EBV DNA in plasma was determined by real-time polymerase chain reaction (PCR). Patients with EBV DNA values ≥400 copies/ml were assigned to the EBV DNA (+) group and patients with EBV DNA values <400 copies/ml were assigned to the EBV DNA (-) group.

A total of 330 patients who met the inclusion criteria underwent plasma EBV DNA tests before treatment, and 112 were positive. Among them, there were 2 cases of stage II, 24 cases of stage III, and 86 cases of stage IV. To incorporate EBV DNA into the prognostic model, missing data had to be interpolated. The interpolation of EBV DNA missing data was performed using the multiple substitutions in chained equations (MICE) method of random forest (parameters setting: random state=10, iterations=20). Training and validation cohorts showed no significant differences in EBV DNA based on the interpolated data.

|  | Training cohort  (n=323) | Validation cohort  (n=139) | *P* |
| --- | --- | --- | --- |
| EBV (before) |  |  | 0.664 |
| Positive | 76 (23.5%) | 36 (25.9%) |  |
| Negative | 153 (47.4%) | 65 (46.8%) |  |
| None | 94 (29.1%) | 38 (27.3%) |  |

|  | Training cohort  (n=323) | Validation cohort  (n=139) | *P* |
| --- | --- | --- | --- |
| EBV (after) |  |  | 0.987 |
| Positive | 102 (31.6%) | 44 (31.7%) |  |
| Negative | 221 (68.4%) | 95 (68.3%) |  |


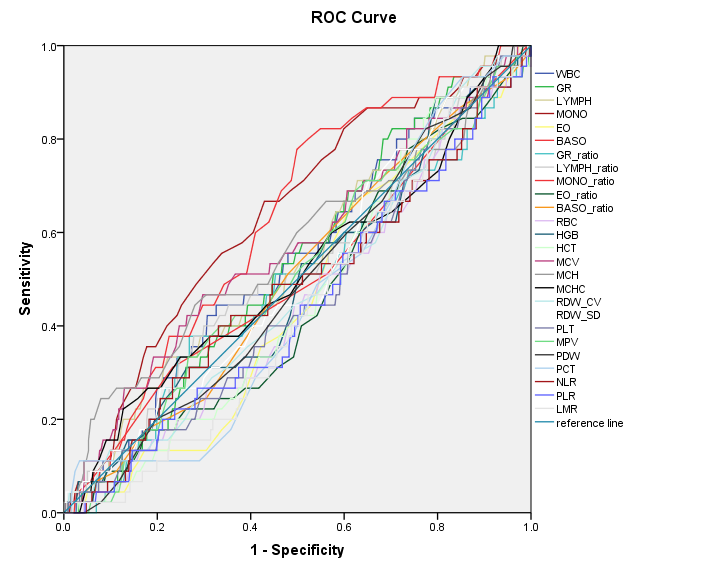


**Figure 2** The ROC curves of blood parameters.

Abbreviation: BASO, basophils; BASO%, ratio of basophils; EO, eosinophils; EO%, ratio of eosinophils; GR, neutrophilic granulocytes; GR%, ratio of neutrophilic granulocytes; HCT, hematocrit; HGB, hemoglobin; LMR, lymphocyte to monocyte ratio; LYMPH, lymphocytes; LYMPH%, ratio of lymphocytes; MCH, mean corpuscular hemoglobin; MCHC, mean corpuscular hemoglobin concentration; MCV, mean corpuscular volume; MONO, monocytes; MONO%, ratio of monocytes; MPV, mean platelet volume; NLR, neutrophil to lymphocyte ratio; PCT, plateletcrit; PDW, platelet distribution width; PLR, platelet to lymphocyte ratio; PLT, platelets; RBC, red blood cells; RDW-CV, variation of RBC distribution width; RDW-SD, standard deviation of RBC distribution width; WBC, white blood cells.

# Table 1 The values of area under curve from blood parameters.

| Blood Parameters | AUC | P Value | 95% CI | |
| --- | --- | --- | --- | --- |
|  |  |  | lower limit | upper limit |
| WBC | .538 | .405 | .449 | .626 |
| GR | .531 | .490 | .448 | .615 |
| LYMPH | .540 | .382 | .452 | .627 |
| MONO | .637 | .002 | .555 | .720 |
| EO | .457 | .340 | .377 | .537 |
| BASO | .506 | .892 | .413 | .599 |
| GR% | .488 | .785 | .396 | .580 |
| LYMPH% | .516 | .721 | .426 | .607 |
| MONO% | .626 | .005 | .549 | .703 |
| EO% | .454 | .309 | .369 | .539 |
| BASO% | .504 | .934 | .417 | .590 |
| RBC | .448 | .249 | .362 | .533 |
| HGB | .493 | .878 | .405 | .581 |
| HCT | .459 | .368 | .379 | .539 |
| MCV | .568 | .132 | .475 | .661 |
| MCH | .565 | .151 | .468 | .662 |
| MCHC | .515 | .747 | .421 | .608 |
| RDW_CV | .477 | .615 | .391 | .564 |
| RDW_SD | .505 | .910 | .418 | .592 |
| PLT | .459 | .361 | .373 | .544 |
| MPV | .509 | .850 | .421 | .596 |
| PDW | .486 | .762 | .403 | .570 |
| PCT | .455 | .324 | .375 | .536 |
| NLR | .482 | .691 | .390 | .574 |
| PLR | .443 | .208 | .356 | .530 |
| LMR | .438 | .169 | .359 | .517 |

# Methods 5 The formula for calculating the rad-score

Rad-score =

0.027377297$\times$CET1-w_Log-sigma-5-0-mm_glrlm_HighGrayLevelRunEmphasis

$-$0.20519048$\times$CET1-w_wavelet-LLH_glcm_ClusterShade

$+$0.06997839$\times$CET1-w_wavelet-LLH_glszm_GrayLevelNonUniformity

$-$0.00815256$\times$CET1-w_wavelet-HHL_glcm_Correlation

$+$0.00917191$\times$CET1-w_wavelet-HHH_firstorder_Mean

$+$0.05779594$\times$CET1-w_wavelet-HHH_gldm_LargeDependenceHighGrayLevelEmphasis

$+$0.006281596$\times$T2-w_log-sigma-4-0-mm_firstorder_Maximum

$+$0.131747741$\times$T2-w_wavelet-HHL_firstorder_Maximum

$-$0.14929471$\times$T2-w_wavelet-HHL_glcm_InverseVariance
